# Hepatitis B Virus X Upregulates HuR Protein Level to Stabilize HER2 Expression in Hepatocellular Carcinoma Cells

**DOI:** 10.1155/2014/827415

**Published:** 2014-02-27

**Authors:** Chao-Ming Hung, Wei-Chien Huang, Hsiao-Lin Pan, Pei-Hsuan Chien, Chih-Wen Lin, Lei-Chin Chen, Yu-Fong Chien, Ching-Chiao Lin, Kar-Hee Leow, Wen-Shu Chen, Jhen-Yu Chen, Chien-Yi Ho, Pao-Sheng Hou, Yun-Ju Chen

**Affiliations:** ^1^Department of General Surgery, E-Da Hospital/I-Shou University, Kaohsiung 824, Taiwan; ^2^School of Medicine for International Students, I-Shou University, Kaohsiung 824, Taiwan; ^3^Center for Molecular Medicine, China Medical University Hospital, Taichung 404, Taiwan; ^4^Graduate Institute of Cancer Biology, China Medical University, Taichung 404, Taiwan; ^5^The Ph.D. Program for Cancer Biology and Drug Discovery, China Medical University, Taichung 404, Taiwan; ^6^Department of Biotechnology, Asia University, Taichung 413, Taiwan; ^7^Department of Medical Research, E-Da Hospital/I-Shou University, Kaohsiung 824, Taiwan; ^8^Department of Medicine and Department of Health Examination, E-Da Hospital/I-Shou University, Kaohsiung 824, Taiwan; ^9^Graduate Institute of Medicine, College of Medicine, Kaohsiung Medical University, Kaohsiung 807, Taiwan; ^10^Department of Nutrition, I-Shou University, Kaohsiung 824, Taiwan; ^11^Department of Biological Science & Technology, I-Shou University, No. 8 Yida Road, Kaohsiung 824, Taiwan; ^12^Department of Family Medicine, China Medical University Hospital, Taichung 404, Taiwan

## Abstract

Hepatitis B virus- (HBV-) associated hepatocellular carcinoma (HCC) is the most common type of liver cancer. However, the underlying mechanism of HCC tumorigenesis is very complicated and HBV-encoded X protein (HBx) has been reported to play the most important role in this process. Activation of downstream signal pathways of epidermal growth factor receptor (EGFR) family is known to mediate HBx-dependent HCC tumor progression. Interestingly, HER2 (also known as ErbB2/Neu/EGFR2) is frequently overexpressed in HBx-expressing HCC patients and is associated with their poor prognosis. However, it remains unclear whether and how HBx regulates HER2 expression. In this study, our data showed that HBx expression increased HER2 protein level via enhancing its mRNA stability. The induction of RNA-binding protein HuR expression by HBx mediated the HER2 mRNA stabilization. Finally, the upregulated HER2 expression promoted the migration ability of HBx-expressing HCC cells. These findings deciphered the molecular mechanism of HBx-mediated HER2 upregulation in HBV-associated HCC.

## 1. Introduction

Hepatocellular carcinoma (HCC) accounts for the majority of liver cancer. The mechanism underlying HCC tumorigenesis involves several etiological factors, and chronic viral infection is the most critical mediator [1, 2]. Hepatitis B virus (HBV) infection is of particular importance for HCC development since the occurrence of over half of HCC cases is associated with its chronic infection [3, 4]. So far, the mechanism of HBV-associated HCC is still not understood completely yet. HBV may mediate HCC formation directly due to the viral inflammation process. But accumulating evidence shows that HBV-encoded regulatory proteins directly contribute to the HCC tumor progression [5]. HBV-encoded X protein (HBx), one of these regulatory proteins, has been reported to play the most significant role in this regulation [6, 7]. Although HBx is a relatively small protein with 154 amino acids, it has diverse functions in both the cytoplasm and nucleus. In the nucleus, it can turn on gene expressions that are important to tumor progression by interacting with transcription factors. In the cytoplasm, it works through activation of RAF/MEK/ERK and PI3K-Akt signaling pathways [6, 8, 9] which are critical downstream effectors of HER receptor tyrosine kinases (RTKs) family [10]. Furthermore, HBx can regulate protein stability via interacting with proteasome subunits [11, 12]. More recently, it is reported to fine-tune gene levels by regulating microRNA (miRNA/miR) expressions [13].

HER family (also known as EGFR/ErbB family) comprises HER1-4 proteins and its activation plays pivotal roles in the regulation of cell growth and survival. Under normal condition, the activation of HER family proteins is strictly controlled by ligand-mediated endocytic degradation. However, once its expression is dysregulated, tumorigenesis may occur. Therefore, overexpression of HER family proteins is frequently observed in many solid tumors, including HCC [14]. Notably, upregulation of HER2 protein (also known as Neu/ErbB2/EGFR2) in HCC has been reported to be associated with HBV infection [15]. More importantly, elevated HER2 protein expression is also found in HCC tumors with HBx expression and is associated with the poor prognosis of HCC patients [16]. However, it remains unclear whether and how HBx regulates HER2 protein expression. As for the regulation of HER2 expression in tumors, several models have been proposed. The HER2 mRNA may be upregulated either by gene amplification or by promoter activation [17, 18]. On the other hand, regulations by RNA-binding protein HuR (also known as Elavl1) or by microRNAs have been reported to contribute to the stabilization of HER2 mRNA [19, 20]. Moreover, the stability of HER2 protein can also be enhanced at posttranslational level [21, 22].

In this study, we demonstrated that HBx increased HER2 protein expression via enhancing its mRNA stability. The induction of HuR expression by HBx contributed to the elevation of HER2 expression, which subsequently rendered HCC cells more metastatic. Our data provided the plausible molecular mechanism of HER2 upregulation by HBx in HBV-associated HCC tumors.

## 2. Materials and Methods

### 2.1. Cell Culture and Reagents

The human hepatocellular carcinoma Hep3B, HepG2, and their HBx-expressing derivatives were cultured and maintained in Dulbecco's Modified Eagle Medium: Nutrient Mixture F-12 (DMEM/F12) supplemented with 10% fetal bovine serum. We purchased antibodies against HER2, EGFR, and HuR as well as bortezomib from Santa Cruz (Santa Cruz, CA). The antibody against HBx was from either Abcam (Cambridge, UK) or GeneTex (Irvine, CA). The antibodies against myc-tag and Tubulin, MG132, Actinomycin D as well as the validated siRNAs for negative control, HBx, HER2, and HuR were all purchased from Sigma-Aldrich (St. Louis, MO). Transfection reagents of DharmaFECT1 and TransIT-2020 were from Dharmacon (Lafayette, CO) and Mirus Bio LLC (Madison, WI), respectively. The QuickGene RNA cultured cell kit was from Kurabo (Osaka, JP). The RevertAid H Minus First Strand cDNA synthesis kit was purchased from Thermo Fisher Scientific (Waltham, MA). The VeriQuest Fast SYBR Green qPCR Master Mix was from Affymetrix (Cleveland, OH). Transwell chambers (24-well insert; pore size, 8 *μ*m) were purchased from Costar Corp. (Cambridge, MA).

### 2.2. Transfection Assay

For plasmid transfection, cells with 60–80% of confluence in a 3.5 cm dish were transfected with 1 *μ*g of myc-HBx expression vector by using 1 *μ*L of TransIT-2020 transfection reagent according to the manufacturer's instruction. Forty-eight hours later, whole cells lysates or mRNAs were harvested and subjected to indicated experiments. For siRNA transfection, cells with 60–80% of confluence in a 3.5 cm dish were transfected with siRNA at final concentration of 100 nM by using 3 *μ*L of DharmaFECT 1 or 1 *μ*L of TransIT-2020 transfection reagent according to the manufacturer's instruction. After 4 days, whole cells lysates or mRNAs were harvested and subjected to further experiments.

### 2.3. Reverse Transcription-Quantitative Polymerase Chain Reaction (RT-qPCR)

The QuickGene RNA cultured cell kit was used for total RNA extraction and the procedure was performed according to manufacturer's instruction. One *μ*g of RNA was applied to reverse transcription by using the RevertAid H Minus First Strand cDNA synthesis kit. The qPCR analysis of HER2 mRNA expressions was performed on Illumina Eco system (Bio-genesis Technologies Inc.) by using VeriQuest Fast SYBR Green qPCR Master Mix and was normalized to actin expression. Student's *t*-test was used to assess the statistical significance.

### 2.4. mRNA Stability Assay

For examination of mRNA stability, cells were first treated with 5 *μ*M Actinomycin D, followed by extraction of total RNA at indicated time point. The extracted RNA was subjected to RT-qPCR and HER2 mRNA stability was in turn determined and quantified. Student's *t*-test was used to assess the statistical significance.

### 2.5. Cell Growth Assay

Cell growth was measured by crystal violet staining assay. Cells with previous treatment were seeded in a density of 1 × 10^4^ and 5 × 10^4^ in each group and allowed to grow. Five days later, relative cell amounts were determined by crystal violet staining. Briefly, cells were washed with 1X PBS once, followed by fixation and staining with 1% crystal violet in a solvent of 30% ethanol for 15–30 minutes at room temperature. Then, cells were washed with tape water till complete elimination of the background interference.

### 2.6. Cell Migration Assay

Cell migration ability was determined by Transwell migration assay with using Transwell chambers. Cells (5 × 10^4^ per well) with previous experimental conditions were seeded on the noncoated membrane of the upper chamber [23]. After 48-hour incubation, cells were washed with 1X PBS once and fixed by 4% formaldehyde for 30 minutes. Then, cells were washed with 1X PBS once again and stained with 1% crystal violet in a solvent of 30% ethanol for 15–30 minutes at room temperature. Cells remaining on the upper chamber were removed by using cotton swab. The number of cells migrating through the pores to the opposite side of the membrane was shown under microscope. Then, the membrane stained by crystal violet was torn out and dissolved in 33% glacial acetic acid overnight. The migrated cell number was quantified by determining the absorbance of OD570. Student's *t*-test was used to assess the statistical significance.

## 3. Results

### 3.1. HBx Expression Was Responsible for the Increase of HER2 Protein Level in HCC Cells

**Figure 1:**
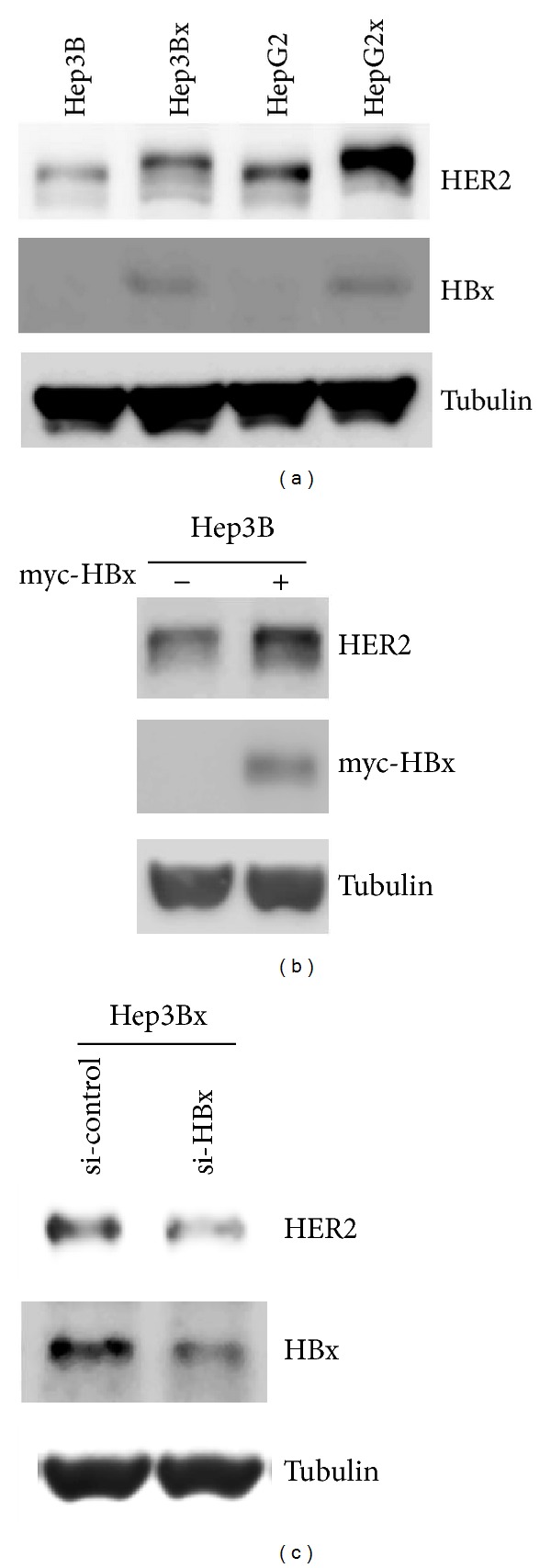
HBx induced HER2 protein expression in HCC cells. (a) The protein expressions of HER2, HBx, and Tubulin in two HBx-paired HCC cells were examined by Western blot (*N* = 4). (b) Myc-HBx expression vector was transiently transfected into Hep3B HCC cells for 48 hrs. The HER2 and myc-HBx protein expressions were analyzed by Western blot (*N* = 3). (c) Transient transfection of HBx siRNA was performed in Hep3Bx cells for 4 days. The HER2 protein expression and gene silencing of HBx protein expression were examined by Western blot (*N* = 3).

To study the association between HBx and HER2 expressions in HCC, two HCC cell lines and their derivatives with stable HBx expression were employed to examine the expression patterns of HER2 in these cells. Consistent with the previous observation in human HCC specimens [16], HER2 protein expression was higher in HBx-expressing Hep3Bx and HepG2x HCC cells than in their Hep3B and HepG2 counterparts (Figure 1(a)). To further demonstrate that the increase in HER2 protein level was caused by HBx expression, the effect of HBx overexpression and gene silence on HER2 expression was examined. We found that enforced expression of HBx into Hep3B cells resulted in the significant increase of endogenous HER2 protein expression (Figure 1(b)). On the contrary, the endogenous HER2 protein expression in Hep3Bx cells was decreased when the HBx expression was silenced (Figure 1(c)). Taken together, these results suggest that HBx expression is responsible for the increase of HER2 protein level in HCC cells.

### 3.2. HBx Increased HER2 Protein Expression by Stabilizing HER2 mRNA in HCC Cells

**Figure 2:**
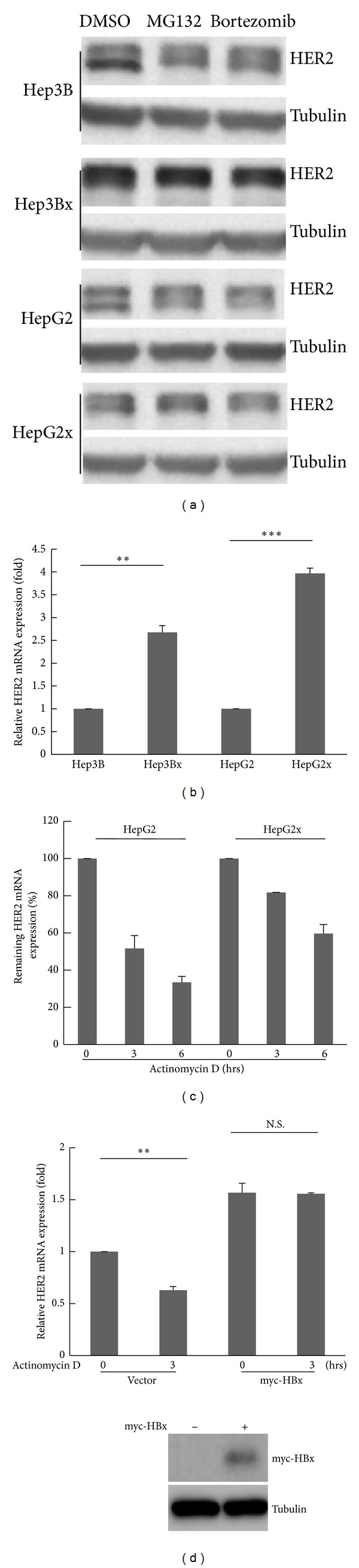
The HER2 mRNA expression was stabilized in HBx-expressing HCC cells. (a) The two HBx-paired HCC cells were treated with proteasomal inhibitors (MG132 and bortezomib) for 24 hrs. The HER2 protein expression was analyzed by Western blot (*N* = 3). (b) The HER2 mRNA expression in two HBx-paired HCC cells was examined by RT-qPCR. The HER2 mRNA expression was normalized to actin expression. Statistical analysis was performed by Student's *t*-test. **P < 0.01; ***P < 0.001 as compared to each control group (*N* = 3). (c) The HepG2 and HepG2x HCC cells were treated with 5 *μ*M Actinomycin D for indicated time periods. The relative remaining HER2 mRNA expression in each of the HCC cells was determined by RT-qPCR. The HER2 mRNA expression was normalized to actin expression (*N* = 4). (d) Hep3B HCC cells were transiently transfected with myc-HBx expression vector for 48 hrs, followed by treatment of 5 *μ*M Actinomycin D. The relative remaining HER2 mRNA expression in each group was determined by RT-qPCR. The HER2 mRNA expression was normalized to actin expression. The protein expression of myc-HBx was confirmed by Western blot. Statistical analysis was performed by Student's *t*-test. **P < 0.01 as compared to each control group. N.S. denoted “not significant” (*N* = 4).

Next, we explored how HBx regulates HER2 expression. Since it has been reported that prolyl isomerase Pin1 is able to maintain the protein stability of HER2 by attenuating its ubiquitin-dependent degradation [24], we first examined whether HBx increased HER2 expression through regulation of its protein stability. To this end, proteasomal inhibitors, including MG132 and bortezomib, were used. As shown in Figure 2(a), HER2 protein expression was not changed in response to treatments with DMSO or proteasomal inhibitors in both parental (Hep3B and HepG2) and HBx-expressing HCC (Hep3Bx and HepG2x) cells, indicating that HBx does not regulate HER2 expression at posttranslational level. We further examined the HER2 mRNA level in these two pairs of HCC cells. We observed that the mRNA expression of HER2 was higher in both Hep3Bx and HepG2x cells as compared to their respective parental cells (Figure 2(b)). It is known that both synthesis and stability contribute to the mRNA expression. We thus clarified whether HBx affects the mRNA stability of HER2 in HCC cells. HepG2 and HepG2x cells were treated with a transcriptional inhibitor Actinomycin D to block the mRNA biosynthesis, and then HER2 mRNA levels were examined after 3 and 6 hrs of treatments. Interestingly, the HER2 mRNA in HepG2x cells was more stable than in HepG2 counterpart (Figure 2(c)), implying that HBx may stabilize the mRNA expression of HER2. To confirm this hypothesis, HCC cells were enforced with HBx expression, followed by Actinomycin D treatment. As shown in Figure 2(d), when HBx was expressed in Hep3B cells (lower panel), HER2 mRNA was present in a more stable state (upper panel). Altogether, these results suggest that HBx increases HER2 protein expression by stabilizing its mRNA in HCC cells.

### 3.3. HBx Upregulated RNA-Binding Protein HuR to Increase HER2 mRNA Stability in HCC Cells

**Figure 3:**
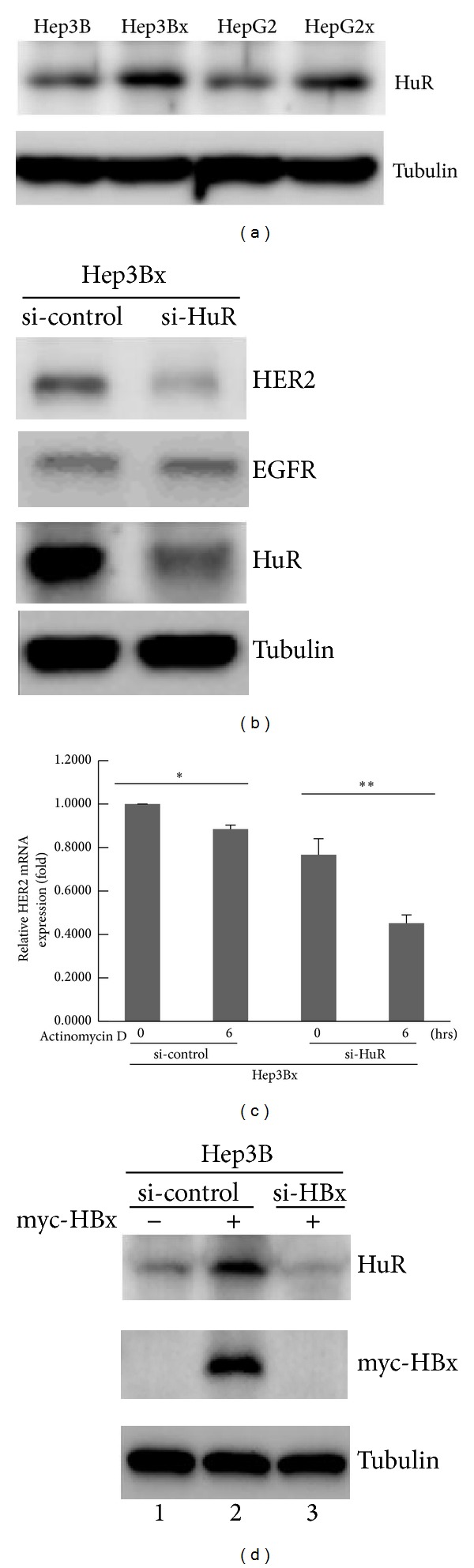
HBx increased HER2 protein expression by HuR-dependent mRNA stabilization in HCC cells. (a) The HuR protein expression in HBx-paired HCC cells was examined by Western blot (*N* = 4). (b) Hep3Bx cells were transiently transfected with either si-control or si-HuR for 4 days. The protein expressions of HER2, EGFR, and HuR were analyzed by Western blot (*N* = 3). (c) Hep3Bx cells were transiently transfected with either si-control or si-HuR for 4 days, followed by the treatment of 5 *μ*M Actinomycin D. The relative remaining HER2 mRNA expression in each group was determined by RT-qPCR. The HER2 mRNA expression was normalized to actin expression. Statistical analysis was performed by Student's *t*-test. *P < 0.05; **P < 0.01 as compared to each control group (*N* = 3). (d) Transient transfection of HBx siRNA was performed in Hep3B cells for 48 hrs, followed by overexpression of myc-HBx expression vector for another 48 hrs. Whole cell lysates were harvested for the examination of HuR and myc-HBx protein expressions by Western blot (*N* = 4).

Next, the molecular mechanism underlying the regulation of HER2 mRNA stability by HBx was further pursued. It is well known that HuR is a ubiquitously expressed RNA-binding protein and is responsible for the mRNA stabilization of many genes, including HER2 and cyclooxygenase-2 (COX2) [25, 26]. Furthermore, it is reported to be involved in the human diseases, including cancers [19, 27]. Here, we investigated whether HuR plays a role in the upregulation of HER2 expression by HBx. The results showed that HuR protein expression was significantly increased in Hep3Bx cells as compared to the Hep3B counterpart. Similar result was also observed in HepG2 and HepG2x cells (Figure 3(a)). Accordingly, HuR siRNA was used to further address whether the upregulated HuR protein expression contributes to the stabilization of HER2 mRNA expression by HBx. As shown in Figure 3(b), when HuR protein expression in Hep3Bx cells was silenced by HuR siRNA, HER2 but not EGFR (epidermal growth factor receptor) protein expression was decreased in parallel, suggesting that the HuR-mediated regulation by HBx is specific for HER2 expression. In consistence with this result, the HER2 mRNA in Hep3Bx cells was degraded faster when cells were transfected with HuR siRNA (Figure 3(c)), indicating that HuR plays a critical role in the stabilization of HER2 mRNA expression. To establish the causal relationship between HuR-mediated mRNA stabilization and HBx-enhanced HER2 expression, the effects of both overexpression and silencing of HBx on HuR expression were examined. We observed that HuR protein expression was significantly induced in Hep3B cells in response to myc-HBx enforced expression (Figure 3(d), compared *lane 2* with *lane 1*). However, the upregulated HuR protein expression was further attenuated when myc-HBx expression was depleted by siRNA (Figure 3(d), compared *lane 3* with *lane 2*). Collectively, these results indicate that HBx protein upregulates HuR expression to stabilize HER2 mRNA, in turn leading to the increase of HER2 protein expression.

### 3.4. The Enhanced Migration Ability of HBx-Expressing HCC Cells Was Attributed to Upregulated HER2 Expression

**Figure 4:**
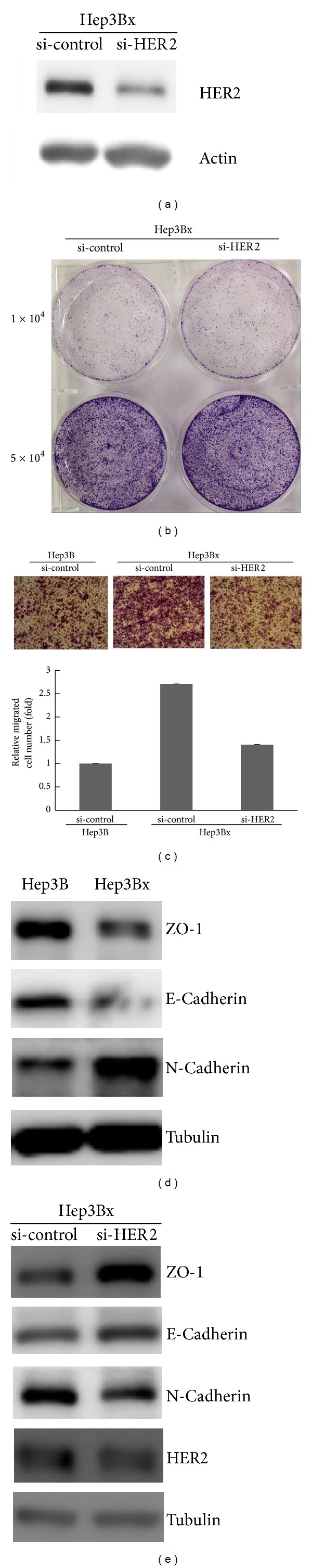
The increased HER2 protein expression was responsible for the migration ability of HBx-expressing HCC cells. ((a), (b), (c), and (e)) Hep3Bx cells were transiently transfected with either si-control or si-HER2 for 4 days. Then, cells were either harvested or reseeded for further experiments. Gene silence of HER2 expression was confirmed by Western blot (a) (*N* = 3). The relative growth rate was determined by crystal violet staining (b) (*N* = 3). The migration of Hep3Bx cells was examined by Transwell migration assay for 48 hrs. The representative pictures of migrated cells were visualized and quantified (c) (*N* = 3). The expressions of metastatic factors were examined by Western blot (e) (*N* = 3). (d) The expressions of metastatic factors in both Hep3B and Hep3Bx cells were examined by Western blot (*N* = 3).

We next investigated the functional roles of increased HER2 protein level in response to HBx expression. Since HER2 is an important oncogene in regulating tumor progression, including tumor growth and metastasis [28], the effect of HBx-increased HER2 expression on cell growth was examined. To this end, deprivation of HER2 expression by siRNA was performed and confirmed (Figure 4(a)). Regardless of the cell density of seeding, silence of HER2 by siRNA did not significantly affect the cell number of HBx-expressing Hep3Bx cells (Figure 4(b)), implying that the cell growth of HBx-expressing cells was not driven mainly by HER2. Our previous study indicated that HBx expression renders HCC cells more metastatic in an Akt/nuclear IKK-*α*-dependent manner [23]. Since HER2 is also known to induce Akt activation and cell metastasis [29], we next investigated whether the increased HER2 expression mediated HBx-enhanced cell migration. As shown in Figure 4(c), the level of cell migration of Hep3Bx cells was obviously less when HER2 expression was silenced by siRNA. To further support this observation, we examined whether this regulation involves an EMT process. It is known that EMT (epithelial-to-mesenchymal transition) is characterized by loss of ZO-1 and E-cadherin and increase of N-cadherin [30]. As shown in Figure 4(d), we found that the expressions of ZO-1 and E-cadherin were decreased whereas N-cadherin expression was increased in Hep3Bx cells, which is correlated to the increased migration ability of Hep3Bx cells. However, this effect was reversed when HER2 protein was silenced (Figure 4(e)). Taken together, these results suggest that HBx protein enhances the migration of HCC cells at least in part through increasing HER2 protein expression.

## 4. Discussion

Gene amplification of the pivotal oncogene HER2 is frequently observed in 20–30% of breast cancer patients and is associated with the disease aggressiveness and poor prognosis [31]. Therefore, HER2 is a rationale target for cancer therapy in those patients. Indeed, HER2 targeted therapies, including monoclonal antibody (trastuzumab) and tyrosine kinase inhibitor (lapatinib), bring promising benefits to breast cancer patients and prolong their overall survival [32–34]. Since there is no effective strategy for HCC therapy so far, many efforts are made to identify the potential oncogenic drivers in HCC and HER2 is one of such potential candidates. However, the results are controversial. Some reports show that HER2 overexpression is uncommon in HCC [35, 36]. In contrast, although HER2 gene amplification is less observed in HCC, several lines of evidence indicate that HER2 protein is overexpressed and plays roles in some HCC cases [37–40]. Notably, it is reported that upregulation of HER2 protein in HCC is found in HCC with HBx expression and is associated with poor prognosis of HCC patients [15, 16]. In consistence with these findings, our data also provided the evidence that HBx is indeed responsible for the upregulation of HER2 protein expression in this study (Figure 1). The investigation of molecular mechanism revealed that HBx increased HuR protein expression to stabilize the mRNA stability of HER2 (Figures 2-3). Furthermore, our unpublished results showed that HBx-expressing HCC cells exhibit higher level of Ser10 phosphorylation of histone H3, an indicator for the transcriptional activity, in HER2 promoter regions. Therefore, the possibility of HER2 promoter activation by HBx still cannot be excluded and needs further investigation. The increased HER2 protein expression rendered HBx-expressing HCC cells more metastatic without affecting their cell growth rate (Figure 4), which may provide a plausible explanation for the poor prognosis of HCC patients with HBx expression [16]. Based on these studies, upregulation of HER2 protein was observed in HCC with HBV infection, especially with detectable HBx expression. Therefore, targeting HER2 in such subgroup of HCC patients may be an appropriate and effective strategy for HCC therapy, which awaits further studies to approve.

In our previous study, HBx-expressing HCC cells were shown to have higher migration ability. The nuclear translocation of IKK-*α* by Akt-dependent ubiquitination to mediate gene expression accounts for the underlying molecular mechanism [23, 41]. HER2 has also been found to increase IKK-*α* nuclear translocation in our previous study [23]. Therefore, it is possible that HBx-increased HER2 expression may enhance cell migration of HCC cells via increasing Akt activity and subsequent nuclear translocation of IKK-*α*. In addition to IKK-*α*, HBx could activate IKK-*β*/TSC-1/mTOR signaling to enhance HCC progression [42]. Furthermore, it is also reported that HBx increases *β*-catenin expression through ERK-dependent GSK-3*β* inactivation [43]. Therefore, further investigations are required to examine whether HER2 also regulates the HBx-dependent HCC progression through these pathways.

In this study, we identified RNA-binding protein HuR as a new target of HBx and also uncovered another pleiotropic role of HBx in the mRNA stabilization (Figures 2-3). The mechanism underlying HuR regulation by HBx is still largely unknown, which needs further studies for clarification. In fact, growing evidence recently indicates that HBx could regulate gene expressions in a microRNA-dependent manner [13, 44–46]. It is known that microRNAs inhibit protein translation by targeting on the 3′ untranslated region (3′UTR) of mRNA. It seems that HBx could simultaneously regulate expressions of both HuR and microRNAs with opposing functions. It will be interesting to investigate how HBx fine-tunes the gene expression by integrating the effects of HuR and microRNAs [47, 48]. It is worth mentioning that our previous report indicates that HBx downregulates EGFR (also known as (HER1/ErbB1) expression in a miR-7-dependent manner [45], whereas the current study shows that HBx upregulates HER2 expression in a HuR-dependent manner. The possibility that HBx exerts such effects to render HCC cells more addicted to HER2 signaling is being investigated.

## 5. Conclusion

This study provides the evidence that HBx protein upregulates HER2 expression through HuR-dependent mRNA stabilization. Thus, HBx-expressing HCC cells exhibit the higher migration ability. Our findings not only clarified the mechanism underlying the HER2 upregulation by HBx, but also demonstrated HuR as a novel target of HBx, implying another pleiotropic function of HBx in the regulation of mRNA stability.

## List of Abbreviations

HBV: Hepatitis B virus
HCC: Hepatocellular carcinoma
HBx: HBV-encoded X protein
EGFR: Epidermal growth factor receptor
RTKs: Receptor tyrosine kinases
miR/miRNA: MicroRNA
COX-2: Cyclooxygenase-2.
